# Early Identification of Prolonged QT Interval for Prevention of Sudden Infant Death

**DOI:** 10.3389/fped.2021.704580

**Published:** 2021-07-29

**Authors:** Georgia Sarquella-Brugada, Oscar García-Algar, María Dolores Zambrano, Anna Fernández-Falgueres, Sebastian Sailer, Sergi Cesar, Giorgia Sebastiani, Julio Martí-Almor, Esther Aurensanz, Jose Carlos Cruzalegui, Erika Fernanda Merchan, Mónica Coll, Alexandra Pérez-Serra, Bernat del Olmo, Victoria Fiol, Anna Iglesias, Carles Ferrer-Costa, Marta Puigmulé, Laura Lopez, Ferran Pico, Elena Arbelo, Paloma Jordà, Josep Brugada, Ramon Brugada, Oscar Campuzano

**Affiliations:** ^1^Arrhythmias Unit, Institut de Recerca Sant Joan de Déu, Hospital Sant Joan de Déu, University of Barcelona, Barcelona, Spain; ^2^Medical Science Department, School of Medicine, University of Girona, Girona, Spain; ^3^Department of Neonatology, Hospital Clínic-Maternitat, Institut Clinic de Ginecologia, Obstetricia i Neonatología, BCNatal, Barcelona, Spain; ^4^Cardiology Service, Hospital Josep Trueta, University of Girona, Girona, Spain; ^5^Department of Neonatology, Faculty of Medicine, Kepler University Hospital, Johannes Kepler University, Linz, Austria; ^6^Department of Cardiology, Hospital del Mar, Barcelona, Spain; ^7^Cardiovascular Genetics Center, University of Girona-Institut d'Investigació Biomèdica de Girona Dr. Josep Trueta, Girona, Spain; ^8^Centro de Investigación Biomédica en Red de Enfermedades Cardiovasculares, Madrid, Spain; ^9^Arrhythmias Unit, Hospital Clinic, Institut d'Investigacions Biomèdiques August Pi i Sunyer, University of Barcelona, Barcelona, Spain

**Keywords:** sudden cardiac death, long QT syndrome, electrocardiogram, genetics, family segregation

## Abstract

**Introduction:** Long QT syndrome is the main arrhythmogenic disease responsible for sudden death in infants, especially in the first days of life. Performing an electrocardiogram in newborns could enable early diagnosis and adoption of therapeutic measures focused on preventing lethal arrhythmogenic events. However, the inclusion of an electrocardiogram in neonatal screening protocols still remains a matter of discussion. To comprehensively analyse the potential clinical value of performing an electrocardiogram and subsequent follow-up in a cohort of newborns.

**Methods:** Electrocardiograms were performed in 685 neonates within the first week of life. One year follow-up was performed if QTc > 450 ms identified. Comprehensive genetic analysis using massive sequencing was performed in all cases with QTc > 470 ms.

**Results:** We identified 54 neonates with QTc > 450 ms/ <470 ms; all normalized QTc values within 6 months. Eight cases had QTc > 480 ms at birth and, if persistent, pharmacological treatment was administrated during follow-up. A rare variant was identified as the potential cause of long QT syndrome in five cases. Three cases showed a family history of sudden arrhythmogenic death.

**Conclusions:** Our prospective study identifies 0.14% of cases with a definite long QT, supporting implementation of electrocardiograms in routine pediatric protocols. It is an effective, simple and non-invasive approach that can help prevent sudden death in neonates and their relatives. Genetic analyses help to unravel the cause of arrhythmogenic disease in diagnosing neonates. Further, clinical assessment and genetic analysis of relatives allowed early identification of family members at risk of arrhythmias helping to adopt preventive personalized measures.

## Introduction

Sudden arrhythmogenic death syndrome (SADS) is a lethal condition that encompasses several cardiac disorders leading to an unexpected death. Individuals have no prior history of any cardiac disease, their hearts appear normal at autopsy, and often the cause of death remains unsolved (1). SADS currently represents 10–20% of sudden deaths in the pediatric population (2). SADS is often associated with primary inherited electrical diseases, called channelopathies, characterized by pathogenic alterations in genes encoding cardiac ion channels or associated proteins. Given that these diseases are inherited, relatives carrying the same genetic alteration may be at risk of lethal episodes. Because sudden death can be the first manifestation of disease, early identification of relatives at risk is necessary to promote adoption of preventive measures (3). Among the SADS cases, the sudden death of infants <1 year of age is named sudden infant death syndrome (SIDS). Despite most SIDS cases remaining unexplained, nearly 15% of them may actually be caused by Long QT Syndrome (LQTS) (4–7).

LQTS is a rare inherited cardiac channelopathy with an estimated prevalence of 1:2,000 (8–10). It is characterized by a prolonged QT interval on an electrocardiogram (ECG) due to delayed ventricular repolarization in a structurally normal heart. The phenotype can range from asymptomatic individuals to ventricular tachyarrhythmias (*torsade de pointes*) and even sudden cardiac death (SCD), occasionally the first manifestation of the disease (11). Currently, appropriate pharmacological therapies reduce mortality (12). To date, hundreds of pathogenic variants for LQTS have been identified in at least 15 genes, and comprehensive genetic analysis can unravel the cause of disease in 80–85% of all diagnosed cases. Primary genes currently associated with LQTS are *KCNQ1* (LQTS type 1) (30–35%), *KCNH2* (LQTS type 2) (25–30%), and *SCN5A* (LQTS type 3) (5–10%) (13, 14). Current guidelines recommend genetic analysis of the main genes as a feasible, successful, and cost-effective approach (15, 16).

A solid correlation between SIDS and LQTS is widely accepted. Thus, a debate is present in the medical community on whether ECGs should become part of the standard neonatal assessment. Some believe that the use of ECG in neonatal screening could allow early identification of alterations in ECG associated with arrhythmogenic diseases, and help prevent SCD (17). In 2006, Quaglini et al. identified a cost-effective approach after performing a programme of neonatal ECG screening in the first month of life (18). Others do not agree, arguing on the cost-effectiveness as well as psychological implications of a borderline QTc result and especially false positive cases (19) although it is still a topic without a conclusion and under debate, as Saul et al. replied (20). It is true that the ECG interpretation in newborns is difficult, as the QT interval varies with the progression of age and may change within the first week of life (21). To date, few published studies have addressed the value of neonatal screening for prolonged QT (4, 10, 22–24). Our study sheds some light on this ongoing argument by comprehensively investigating QTc results from a prospective 3-year registry in newborns.

## Methods

### Cohort

Our prospective study was conducted during 2016–2018 in two maternity institutes (Hospital Sant Joan de Déu, Hospital Clinic-Maternitat) in Catalonia, Spain. Clinical evaluation of newborns included a complete physical examination (regular neonatal protocol) and 12-lead ECG. Exclusion criteria for newborns: maternal medication or any other confounding factor causing transient QTc prolongation, delivery of the baby in the medical outpatient, transfer of child/mother to an intensive care unit after post-delivery, abortion, maternal age <18 years old, or parents Spanish/English/French language skills inadequate to achieve informed consent.

In all available relatives of newborns with QTc > 470 ms, clinical assessment was performed. At least 12-lead ECG and echocardiogram were performed and, if necessary, an exercise stress test and/or 24-h Holter was also performed. Family members included in our study were clinically evaluated at Hospital Sant Joan de Déu, Hospital Josep Trueta, and Hospital Clinic de Barcelona.

### Clinical Assessment

Twelve-lead ECGs were recorded at a speed of 25 mm/s with a Cardiosoft v.6.7.3 (GE Healthcare®, Spain) within 48 h and at 1 week of life in every participant. Each ECG was analyzed by two independent expert cardiologists. If there was any discrepancy, a further ECG analysis was performed by a third senior cardiologist. The QT interval was measured manually from the onset of the Q wave to the end of the T wave. Bazett's formula was used for diagnosis, as currently recommended by European Society of Cardiology (25). Bazett's correction provides an effective heart rate-independent QTc and accurately identifies neonates affected by LQTS (26). In addition, a recent study concluded that Bazett's formula indicates a stable value during first month of life (27). The end of the T wave was defined as the intersection of a tangent to the steepest slope of the T wave and the baseline (28). This method can lead to an underestimation of the QT interval if there is a double slope on the descending part of the T wave, and this was taken into consideration when measuring the QT interval (29).

No supplementary action was performed in neonates with QTc <450 ms at 1 week of life. Follow-up was conducted for all neonates with QTc ≥ 450 ms, accordingly to published studies (17). Additional ECGs were performed at ages 1 month, 3 months, 6 months, and 1 year old in each case. If any of these follow-up ECGs showed QTc <450 ms, no more action was performed. In neonates with initial QTc of ≥470 ms and persistent at week of life, genetic analysis was performed immediately. In addition, all available relatives of neonates genetically analyzed were clinically assessed. If we found a positive genetic result in a neonate, segregation in all previously clinically assessed relative was performed to obtain a comprehensive genotype–phenotype correlation.

### Genetic Analysis

Genomic DNA was analyzed using next generation sequencing (NGS) technology. We screened 120 genes involved in inherited pathologies associated with SCD (*ABCC9, ACTC1, ACTN2, AKAP9, ANK2, ANKRD1, BAG3, CACNA1C, CACNA2Da, CACNA1G, CACNA1H, CACNA1I, CACNB2, CALM1, CALM2, CALM3, CALR3, CASQ2, CAV3, CRYAB, CSRP3, CTNNA3, GJA1, CTF1, DES, DMD, DMPK, DPP6, DSC2, DSG2, DSP, DTNA, ECE1, EMD, EN1, EYA4, FHL2, FKTN, FLNA, FLNC, GAA, GJA5, GLA, GPD1L, HCN1, HCN2, HCN4, JPH2, JUP, KCNA5, KCND3, KCNE1, KCNE2, KCNE3, KCNE4, KCNE5, KCNH2, KCNJ2, KCNJ5, KCNJ8, KCNQ1, LAMA4, LAMP2, LDB3, LMNA, MYBPC3, MYH6, MYH7, MYL2, MYL3, MYLK2, MYOZ2, MYPN, NEBL, NEXN, NOS1AP, NOTCH1, NPPA, NUP155, PDLIM3, PHOX2A, PHOX2B, PITX2, PKP2, PLN, PRKAG2, RANGRF, RBM20, RYR2, SCN1B, SCN2B, SCN3B, SCN4B, SCN5A, SCN10A, SDHA, SGCD, SLC22A5, SLC6A4, SLC8A1, SLMAP, SLN, SNTA1, TAZ, TCAP, TGFB3, TLX3, TMEM43, TMPO, TNNC1, TNNI3, TNNT2, TP63, TPM1, TRDN, TRIM63, TRPM4, TTN, TTR*, and *VCL*) (30). The panel also included genes encoding structural proteins, as some recent publications have suggested that variants in these genes may be associated with SIDS (31, 32). All gene isoforms described in Ensembl 75 (www.ensembl.org) that have been linked with either a RefSeq code (https://www.ncbi.nlm.nih.gov/refseq/) or CCDS (https://www.ncbi.nlm.nih.gov/projects/CCDS/CcdsBrowse.cgi) were included. Coordinates of sequence data were based on UCSC human genome version hg19 (NCBI GRCh37 built). Biotinylated cRNA probe solution was used as a capture probe (Agilent Technologies, Santa Clara, CA, USA). Probes were designed using eArray (Agilent Technologies).

Secondary bioinformatic analysis of the data included adaptor and low-quality bases trimming on FASTQ files. Trimmed reads were mapped with GEM III. The output was sorted, and uniquely and properly mapped read pairs were selected. Finally, variant calling over the cleaned BAM was performed with SAMtools v.1.2 together with an ad hoc developed script. The final annotation steps provided information included in public databases. Non-common [minor allele frequency (MAF) <1%] genetic variants identified in NGS analysis were confirmed by Sanger sequencing. Exons and exon–intron boundaries of each gene were amplified (Verities PCR, Applied Biosystems, Austin, TX, USA), PCR products were purified (Exosap-IT, Affymetrix Inc., USB Products, Cleveland, OH, USA), and purified products were directly sequenced in both directions (Big Dye Terminator v3.a and 3130XL Genetic Analyzer, both from Applied Biosystems). Posterior SeqScape Software v2.5 (Life Technologies, Carlsbad, CA, USA) analysis was used to compare results with the reference sequence from hg19.

Identified variations were compared with DNA sequences from 400 healthy Spanish individuals (individuals not related to any index case and of the same ethnicity) as control cases and were contrasted with the Human Gene Mutation Database (HGMD) (www.hgmd.cf.ac.uk/ac/index.php), HapMap (https://www.coriell.org/1/NHGRI/Collections/HapMap-Collections/HapMap-Project), 1000 Genomes Project (www.1000genomes.org), Exome Variant Server (EVS) (https://evs.gs.washington.edu/EVS/), and Genome Aggregation Database (gnomAD) (https://gnomad.broadinstitute.org/). Sequence variants were described following HGVS rules (www.hgvs.org) and checked in Mutalyzer (www.mutalyzer.nl). Several *in silico* predictors of pathogenicity were consulted, including Functional ANnotations for Non-Synonymous SNVs- (FannsDB) (https://bbglab.irbbarcelona.org/fannsdb/), Mutation Taster (www.mutationtaster.org), Protein Variation Effect Analyzer (PROVEAN) (http://provean.jcvi.org/index.php), and PolyPhen-2 Polymorphism Phenotyping v2 (PPH2) (http://genetics.bwh.harvard.edu/pph2/). Alignment of DNA sequences for different species was performed for novel variations using Uniprot database (www.uniprot.org). In addition, protein structure and domains were consulted at STRING (www.string-db.org) and SMART databases (http://smart.embl-heidelberg.de/).

Regarding copy number variation (CNV), our approach focuses on capturing significant differences between expected normalized coverage and obtained normalized coverage for a given sample in a region of interest. Several samples were analyzed to corroborate similar levels of coverage between samples, as already published by our group (33). All CNVs were compared with CNV Control database (https://humandbs.biosciencedbc.jp/en/hum0126-v1), Database of Genomic Variants (DGV) (http://dgv.tcag.ca/dgv/app/home), DECIPHER (https://www.deciphergenomics.org/), and gnomAD (www.gnomad.broadinstitute.org). As in punctual and small indels variants, the CNV detection by NGS is also confirmed with an alternative technology. Finally, complete genetic segregation in relatives was performed using the Sanger method. Only rare genetic variants confirmed in the index case were analyzed in all available relatives to perform comprehensive genotype–phenotype family correlation.

Concerning the interpretation of genetic variants, we classified each variant (B, Benign; LB, Likely Benign; VUS, Variant of Unknown Significance; LP, Likely Pathogenic; P, Pathogenic) following current recommendations of the American College of Medical Genetics and Genomics (ACMG) and the Association for Molecular Pathology (AMP) (34–36). For frequency of disease-causing variants, the vast majority of pathogenic variants are extremely rare (<0.01%) (37). In addition, all rare variants were consulted in ClinGen (www.clinicalgenome.org), VarSome (www.varsome.com), CardioClassifier (www.cardioclassifier.org), CardioVAI (www.cardiovai.engenome.com), and CardioBoost (www.cardiodb.org/cardioboost/). To avoid bias, three authors with PhDs in human genetics (OC, MC, RB) independently and comprehensively investigated published genetic data concerning each analyzed variant in our study. In addition, four independent expert cardiologists -OGA, JMA, JB, RB- (including two pediatric cardiologists -GSB, SC-) comprehensively reviewed available clinical data to reconfirm diagnoses following current guidelines (16). All investigators discussed data included in each item of the ACMG/AMP and consensus as well as final classification of all variants.

## Results

### Cohort

Between 2016 and 2018 (both years included) a total of 748 neonates received an ECG in first 48 h after birth and at 1 week of life. Sixty-three newborns with any suspicious or diagnosed prenatal and/or postnatal congenital alterations were excluded to avoid bias. Our cohort included a total of 685 newborns after obtaining written informed consent from parents/tutors. All cases were born at full term with uneventful antenatal and perinatal history. All patients included in our study were Caucasian except 43 (19 North Africa, 15 Asian and 9 Black). Of the newborns, 46.8% were males, with a mean duration of pregnancy of 39.6 weeks, a mean weight of 3.428 g, and a mean length of 50.9 cm.

**Figure 1:**
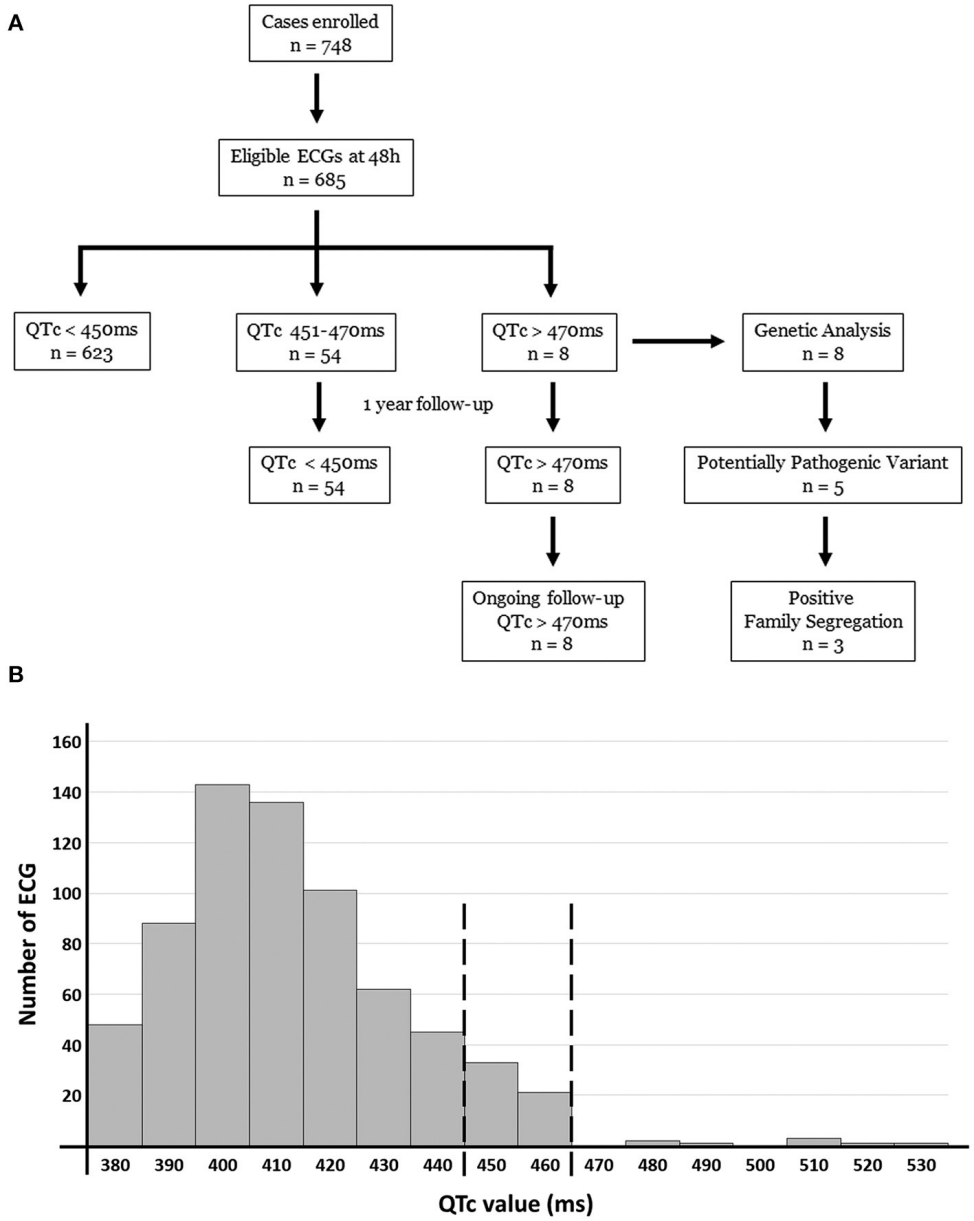
Diagram **(A)** and histogram **(B)** of our study population and results obtained. Of 685 ECG included in our study, 8 cases showed a QTc > 470 ms at birth. All these 8 cases maintain enlarged QTc and are in clinical follow-up so far. Genetic analysis identified 5 of these 8 cases carrying a potential variant as the most plausible cause of LQTS. In 3 families we observed a segregation.

We identified no significant differences between ECGs performed during the first week of life. Therefore, the first QTc value obtained in the first 48 h of life (<450, 450–470 or >470 ms) was similar to register at 1 week. Most ECGs performed in the first 48 h (*n* = 623, 90.94%) revealed a QTc <450 ms, and no follow up was performed after second ECG performed in 1 week of life. At our knowledge, none of these normal QT cases have shown any arrhythmogenic event so far. In 54 neonates (7.88%), the QTc was 450–470 ms at 1 week of life (62.5% males) (Figure 1; Table 1). All these neonates were Caucasian. Neither inherited arrhythmia nor SCD history had been documented in any of these families at the moment of delivery. Clinical follow-up showed QTc normalization in all cases before the first year of life. During follow-up of these 54 cases, no anomalous clinical events have been reported to date (syncope, infection, metabolic disorder, or epileptic episode). Finally, eight neonates (1.16%) (64.5% males, mean duration of pregnancy of 39.1 weeks, mean weight of 3.547 g, and mean length of 51.1 cm) had a QTc interval > 470 ms in first ECG performed (within 48 h and also at 1 week of life). Of them, five cases (three female and two males had a QTc > 500 ms). In all cases, successive ECGs during follow-up confirmed the elongated QTc values already observed on the first ECG. Echocardiogram were performed and no structural heart alterations were identified in any of them.

**Table 1:** QTc intervals in similar studies.

**Origin of cohort**		**Italian (10)**	**Japanese (22)**	**German (23)**	**Catalan**
Years of study		2001–2006	2010–2011	2015–2018	2016–2018
Centers involved		18	8	1	2
Samples analyzed		*n* = 43.080	*n* = 4.285	*n* = 2.251	*n* = 685
Time at first ECG		2–4 weeks	4 weeks	1 week	48 h/1 week
QTc (ms)	>470	31 (0.07%)	5 (0.12%)	62 (2.8%)	8 (1.16%)
	450–470	205 (0.47%)	37 (0.86)	37 (1.6%)	54 (7.88%)
Genetic alteration potentially pathogenic		16 of 42 (38.1%)	1 of 5 (20%)	2 of 12 (16.6%)	5 of 8 (62.5%)
Definite diagnosis of LQTS		17 (0.04%)	1 (0.02%)	2 (0.09%)	1 (0.14%)^*^

*Italian study (10), Japanese study (22), and German study (23). LQTS, long QT syndrome; ECG, electrocardiogram*.

* *If families referred to our center due to the suspicious inherited arrhythmogenic syndrome not included, the percentage decrease to 1 case (0.14%)*.

The European Guidelines recommended to perform ECG screening in the 3rd−4th week of life due to first month is a period of adaptation of the newborn and some suspicious QT prolongations become to be normalized spontaneous (25). At month of life, ECG still showed severe QTc values and then, all cases were treated using propranolol following current recommendations: an initial dose of 0.5–1 mg/kg/day in divided doses every 6–12 h until 5–7 days, and then a standard dose of 1–5 mg/kg/day (maximum 8 mg/kg/day). During follow-up, no anomalous clinical events were reported in any cases, including syncope, infection, metabolic disorder, or epileptic episode. No mothers had documented ingestion of a drug prolonging QT during pregnancy. Of the eight respective mothers, only one (12.5%) experienced at least one previous spontaneous abortion (family 3), and three mothers (37.5%) had a family history of SADS (families 1, 2, and 6). These four families were referred to our center (national reference center for pediatric arrhythmias) due to suspicious potential inherited arrhythmias in relatives. At the moment of the inclusion in our study, no definite diagnosis of LQTS had been reported in any the relatives. Clinical diagnosis and genetic analysis in relatives of these four families were performed after delivery and during the follow-up of the newborn.

### Genetics

Comprehensive genetic analysis was performed in the eight cases with QTc > 470 ms after ECG performed at 1 week of life. For NGS data, an average call rate of 99.85% was achieved at 30x coverage. The median coverage per sample was 860x (715–1105x). An average of seven failed exons occurred in each sample, and all these exons were amplified using Sanger sequencing. All rare variants (MAF <1%) were confirmed also using Sanger sequencing. No CNVs were identified in any genes analyzed.

Genetic analysis identified at least one rare single nucleotide variant (SNV) in each of these eight cases with QTc > 470 ms. A total of 24 SNVs was identified, all confirmed by Sanger sequencing. Most SNVs were in exonic regions; only two were located in intronic regions -*SCN10A*_c.692-4G>A (number 4), and *MYH6_*c.3343-3delC (number 6)-. Thus, of 22 exonic variants, 19 were *missense* (86.36%; 2 novel), two were *indels*, and one was a STOP codon. Five rare variants were novel (20.83%), and eleven (45.83%) were previously reported in HGMD (CM020455, CM40442, and CM1110434) and/or ClinVar (Table 2). Finally, following ACMG guidelines, four of 24 rare variants were classified as B (16.6%), eight as LB (33.3%), and seven as VUS (29.16%). Considering a potential deleterious role, three (12.5%) were classified as LP -*KCNQ1_*p.Ser253Asn (case 1), *KCNQ1*_p.Ala590Thr (case 5), and *GAA_*p.Ala582Val (case 8)- and two (8%) as definite P -*KCNH2_*p.Arg744Ter (case 2), and *RyR2_*p.Val93MetfsTer29 (case 5)- (Figure 1; Table 2).

**Table 2:** Genetic data of variants identified.

**Case**	**Gender**	**First ECG QTc**	**Gene**	**Nucleotide**	**Protein**	**dbSNP**	**gnomAD frequency**	**HGMD (disease)**	**ClinVar (disease)**	**ACMG Score**	**Family history**
1	Female	527 ms	*KCNQ1*	c.757_758delTCinsAA	p.(Ser253Asn)	Novel	NA	NA	NA	LP	LQTS^*^
			*TTN*	c.55406G>A	p.(Arg18469His)	rs775651612	225334/14 (0.006%)	NA	VUS	LB	
2	Male	519 ms	*KCNH2*	c.2230C>T	p.(Arg744Ter)	rs189014161	NA	CM020455 (LQTS)	P (LQTS)	P	SCD/LQTS^*^
3	Male	481 ms	*SCN5A*	c.4655A>T	p.(Gln1552Leu)	rs1387460395	249338/1 (0.0004%)	NA	NA	VUS	SCD/LQTS^*^
			*KCNE1*	c.127G>A	p.(Glu43Lys)	rs755781709	251436/7 (0.002%)	NA	VUS (LQTS)	VUS	
			*CACNA1H*	c.1067A>G	p.(Asn356Ser)	Novel	NA	NA	NA	VUS	
			*NEXN*	c.1238A>G	p.(Gln413Arg)	Novel	NA	NA	NA	LB	
4	Female	535 ms	*SCN10A*	c.692-4G>A	NA	rs373083732	248028/12 (0.004%)	NA	NA	B	LQTS
			*HCN4*	c.1703G>C	p.(Ser568Thr)	rs138714806	276788/263 (0.1%)	NA	VUS (BrS)	B	
			*CTNNA3*	c.1823A>T	p.(Asp608Val)	rs138314889	248484/12 (0.004%)	NA	VUS (ACM)	LB	
			*VCL*	c.1226G>A	p.(Arg409Gln)	rs1018998675	251408/4 (0.001%)	NA	NA	LB	
			*SLC22A5*	c.197C>T	p.(Thr66Ile)	rs1169005119	226870/2 (0.0008%)	NA	NA	VUS	
5	Male	499 ms	*KCNQ1*	c.1768G>A	p.(Ala590Thr)	rs199472813	250944/2 (0.0007%)	CM040442 (LQTS)	LP (LQTS)	LP	SCD
			*RyR2*	c.277_290del	p.(Val93MetfsTer29)	Novel	NA	NA	NA	P	
			*MYBPC3*	c.817C>T	p.(Arg273Cys)	rs551119259	175154/2 (0.001%)	CM1110434 (HCM)	VUS (HCM)	VUS	
			*TTN*	c.61120G>A	p.(Glu20374Lys)	rs923029692	248726/2 (0.0008%)	NA	VUS (DCM)	VUS	
6	Male	485 ms	*ANK2*	c.2863G>C	p.(Asp955His)	rs768070511	250698/2 (0.0007%)	NA	NA	VUS	SCD/LQTS^*^
			*SCN1B*	c.635C>T	p.(Thr212Met)	rs748784203	251418/5 (0.001%)	NA	NA	LB	
			*TMPO*	c.764C>T	p.(Pro255Leu)	rs762263378	276790/8 (0.002%)	NA	VUS	B	
			*MYH6*	c.3343-3delC	NA	rs1027186100	31384/16 (0.006%)	NA	VUS (HCM)	B	
7	Male	510 ms	*SLC8A1*	c.2075A>T	p.(Glu692Val)	rs5557	276598/357 (0.1%)	NA	NA	LB	No
8	Female	514 ms	*ANK2*	c.8936G>C	p.(Gly2979Ala)	rs1217618151	250916/1 (0.0003%)	NA	NA	VUS	No
			*SCN4B*	c.115A>G	p.(Thr39Ala)	rs756210130	251480/8 (0.003%)	NA	VUS (LQTS)	LB	
			*GAA*	c.1745C>T	p.(Ala582Val)	Novel	NA	NA	NA	LP	

*ACM, Arrhythmogenic cardiomyopathy; ACMG, American Colleague of Medical Genetics and Genomics; B, Benign; BrS, Brugada syndrome; DCM, Dilated cardiomyopathy; ECG, Electrocardiogram; HCM, Hypertrophic cardiomyopathy; HGMD, Human Genome Mutation Database; LB, Likely Benign; LP, Likely Pathogenic; LQTS, Long QT syndrome; ms, milliseconds; NA, Not Available; P, Pathogenic; SCD, Sudden cardiac Death; VUS, Variant of Uncertain Significance*.

* *Families referred to our center due to the suspicious inherited arrhythmogenic syndrome*.

### Neonates

**Figure 2:**
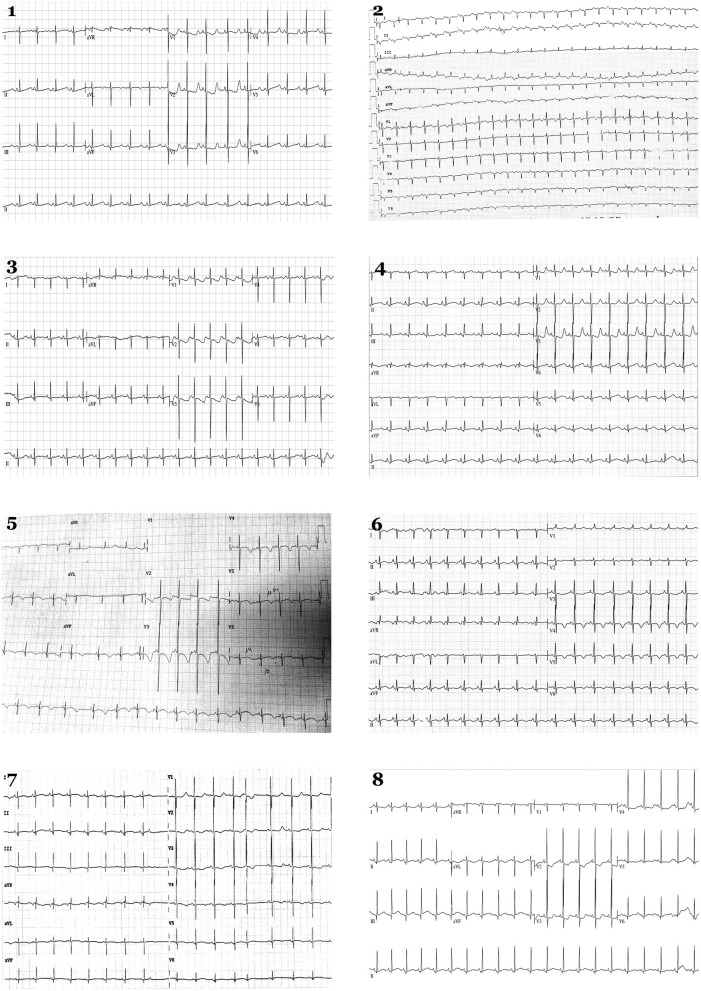
Electrocardiograms in neonates. Eight newborns show a QTc > 480 ms in the first 48 h. Each number corresponds to index case of each family analyzed.

Eight cases (1.16%) showed a definite QTc > 470 ms and were genetically analyzed (Figures 1, 2). Case 1 had QTc of 527 ms at birth. During follow-up, QTc was continuously >470 ms until propranolol administration and no arrhythmogenic episodes were documented. Genetic analysis identified two rare variants: *KCNQ1_*p.Ser253Asn (novel and classified as LP), and *TTN* _p.Arg18469His (classified as LB). Case 2 had a maximum QTc of 519 ms at birth and kept QTc > 470 ms in follow-up until propranolol treatment, which reduced QTc to near normal values. No arrhythmogenic episodes were reported. Genetic analysis identified the rare variant *KCNH2_*p.Arg744Ter -previously reported as cause of LQTS in HGMD (CM020455)-. This variant is classified as P following ACMG recommendations as well as in ClinVar for LQTS. Case 3 had a maximum QTc of 481 ms at birth. During follow-up, QTc was continuously > 470 ms. Propranolol was administrated, QTc normalized and no arrhythmogenic episodes have been reported to date. He carried four rare variants: *SCN5A*_p.Gln1552Leu (classified as VUS), *KCNE1_*p.Glu43Lys (classified as VUS by ACMG and ClinVar), *CACNA1C*_p.Asn356Ser (classified as VUS), and *NEXN*_p.Gln413Arg (classified as LB following ACMG recommendations). Case 4 had a maximum QTc of 535 ms at birth. All QTc values during follow-up were continuously >470 ms, and propranolol was administrated, QTc almost normalized and no arrhythmogenic episodes have been reported so far. She carried five rare variants: *SCN10A*_c.692-4G>A (classified as B following ACMG recommendations), *HCN4_*p.Ser568Thr (classified as B by ACMG but as VUS in ClinVar for Brugada syndrome), *CTNNA3_*p.Asp608Val (classified as LB by ACMG but as VUS by ClinVar for arrhythmogenic cardiomyopathy), *VCL*_p.Arg409Gln (classified as LB), and *SLC22A5_*p.Thr66Ile (classified as VUS). Case 5 had a QTc of 499 ms at birth plus bradycardia. In follow-up, QTc was continuously >470 ms. Propranolol was administered, QTc value normalized and no arrhythmogenic episodes have been reported to date. He carried four rare variants: *KCNQ1_*p.Ala590Thr [previously reported as a cause of LQTS in HGMD (CM040442), classified as an LP by ACMG, and also as LP by ClinVar for LQTS], *RyR2_*p.Val93Metfs^*^29 (novel and classified as P), *MYBPC3_*p.Arg273Cys [classified in HGMD as potentially deleterious for hypertrophic cardiomyopathy (HCM), classified as VUS by ACMG, and also considered VUS by ClinVar for HCM], and *TTN_*p.Glu20374Lys -classified as VUS by ACMG and also predicted as VUS by ClinVar for dilated cardiomyopathy (DCM)-. Case 6 had a QTc of 485 ms at birth. During follow-up, QTc was continuously >470 ms. Propranolol was administrated to normalize QTc, and no arrhythmogenic episodes have been reported. He carried four rare variants: *ANK2_*p.Asp955His (classified as VUS following ACMG recommendations), *SCN1B*_p.Thr212Met (classified as LB by ACMG), *TMPO*_p.Pro255Leu (classified as B by ACMG and predicted as VUS by ClinVar for HCM), and *MYH6_*c.3343-3delC (classified as B by ACMG and predicted as VUS by ClinVar for HCM). Case 7 had a QTc of 510 ms at birth. In follow-up, QTc was continuously >470 ms. Propranolol was administrated to normalize QTc value, and no arrhythmogenic episodes have been reported so far. He carried only one rare variant, *SLC8A1C*_p.Glu692Val (classified as LB following ACMG recommendations). Case 8 presented QTc of 514 ms at birth, also with >470 ms during follow-up. Propranolol was started, and no arrhythmogenic episodes have been currently reported. She carried three rare variants: *ANK2_*p.Gly2979Ala (classified as LB), *SCN4B*_p.Thr39Ala (classified as LB and predicted as VUS in ClinVar for LQTS), and *GAA_*p.Ala582Val (novel and classified as LP) (Figures 1, 2; Table 2).

### Family Segregation

**Figure 3:**
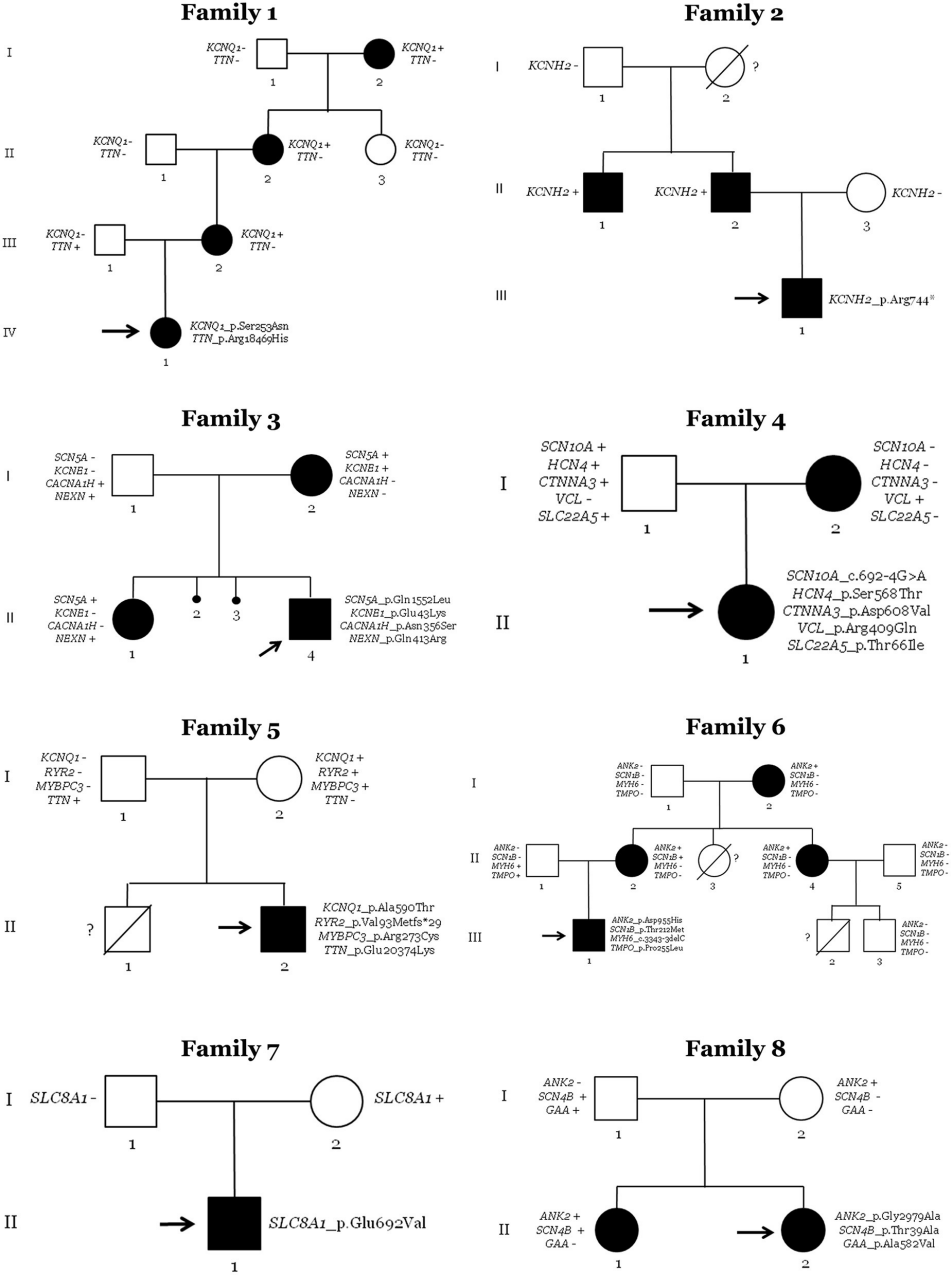
Pedigrees of families. Generations are indicated on the left side. Each individual of direct family lineage is identified with a number. Definite patients diagnosed with Long QT syndrome are shown in black, clinically unaffected patients are in white, and slashes indicate a deceased relative. Index case is indicated with an arrow. Sign plus indicates carrier of the genetic variant. Minus sign indicates not carrier of the genetic variant. Interrogative sign indicates no genetic analysis available.

After NGS analysis in the eight neonates with QTc > 470 ms, genotype–phenotype correlation was performed in all available relatives. A total of 38 people (8 newborns and 30 family members) was clinically assessed and genetically analyzed. In each family, at least the parents of the index case were both clinically and genetically analyzed. Of the genetic variants identified in the index cases, none *de novo*. Nineteen people were clinically affected with LQTS (8 newborns and 11 family members). Four families (50%) showed complete penetrance. Hence, in 5 families (62.5%) we identified a potential cause of the disease, nearly 80% of genetic yield reported in LQTS cohorts (Figures 1, 3) (38).

In the family of case 1, four relatives (all women of four generations) had a diagnosis of LQTS (index case: IV.1; family members: I.2, II.2, and III.2). QTc values of all relatives were also high at diagnosis, but pharmacological treatment practically normalized QTc values. All were currently being treated with antiarrhythmic drugs. Neither syncope nor any malignant arrhythmogenic episode has been documented to date. All clinically diagnosed relatives carried the same rare variant in *KCNQ1*, showing complete penetrance. Other rare variants in *TTN* were identified in the index case. However, family segregation discarded a pathogenic role of *TTN* because only the index case carried this rare variant. However, it is possible that a combination of both rare variants leads to early expression of QTc or a more aggressive phenotype, as previously suggested (39). Therefore, *KCNQ1_*p.Ser253Asn was the most plausible cause of disease in this family. It is important to note that this mother was referred at our center due to suspicious family history of inherited arrhythmia, but no complete assessment of relatives had been performed in other centers before inclusion in our study.

The family of case 2 showed three clinically diagnosed relatives (index case: III.1; family members: II.1 and II.2). Both relatives were men of two different generations with QTc > 470 ms. They were being pharmacologically treated, with no arrhythmogenic episodes documented so far. All clinically affected relatives carried the same rare variant (*KCNH2*_p.Arg744Ter). Hence, this family had complete penetrance. The paternal grandmother (I.2) died suddenly at a young age, but unfortunately no autopsy was performed. Despite no available sample for post-mortem genetic analysis of this case, it is plausible to suggest that she was a carrier of the same rare variant in *KCNH2*_p.Arg744Ter because the paternal grandfather (I.1) did not carry the rare variant. *De novo* does not seem probable due to both brothers (II.1 and II.2) carry the same rare variant. Therefore, the rare variant *KCNH2_*p.Arg744ter [previously associated with LQTS (CM020455) and currently classified as P following ACMG recommendations] seems to be the cause of the disease in this family. As occurred in the first family, mother was referred at our center due to suspicious family history of inherited arrhythmia, but no complete assessment of relatives had been performed previously for inclusion in our study.

The third family had three relatives clinically diagnosed with LQTS (index case: II.4; family members: I.2 and II.1). The index case's sister and mother had QTc values of >470 ms at diagnosis. Pharmacological treatment was ongoing, and no arrhythmogenic episodes were reported. All three carried the same rare genetic variant (*SCN5A*_p.Gln1552Leu). Hence, complete penetrance also occurs in this family. In addition, two previous intra-utero deaths were documented (II.2 and II.3) with no conclusive cause of death after autopsy, suggesting a potential arrhythmogenic cause. No molecular autopsy was performed in either case. It has been reported that mothers with more than one intra-utero deaths may carry a potential pathogenic variant in genes associated with LQTS (40), including *SCN5A* (41). Unfortunately, no DNA is currently available to perform genetic analysis. The presence of another rare variant in *KCNE1* could not be considered the cause of disease due to the absence of this rare variant in one clinically affected relative. The *KCNE1*_p.Glu43Lys variant was classified as VUS and could be a phenotype modifier, although the combination of both rare variants does not seem to induce a different phenotype because there were neither significant differences nor clinical manifestations between relatives carrying only one (*KCNQ1*) or both rare variants (*KCNQ1* + *KCNE1*). Therefore, the most reasonable cause of LQTS seems to be the *SCN5A* variant. This mother was referred at our center due to suspicious family history of inherited arrhythmia potentially associated with previous episodes of abortion. No complete assessment of relatives had been performed in first center before inclusion in our study.

In the fourth family, the newborn (II.1) and her mother (I.2) were clinically diagnosed with LQTS. The mother had a QTc of 472 ms at diagnosis. She was being pharmacologically treated, reducing QTc values to normal levels, and no arrhythmogenic episodes have been documented. After NGS analysis and genotype–phenotype segregation, only one rare variant in *VCL* was identified in both clinically diagnosed cases. The rare variant is classified as a LB, and deleterious alterations in this gene have been associated with DCM. Therefore, no rare variant in any gene currently associated with the disease was identified in this family as a cause of LQTS.

In the fifth family, only the index case (II.2) was diagnosed with LQTS (QTc of 499 ms). A bradycardia was also identified at birth that disappear progressively during follow-up, but QTc value remained pathologic until pharmacological treatment. The index case carries four rare variants in four different genes: *KCNQ1, RyR2, MYBPC3*, and *TTN*. The first three rare variants were inherited from the mother (I.2), and the *TTN* variant was inherited from her father (I.1). The rare variant in *MYBPC3* was previously suggested as pathogenic in HCM cases (CM1110434) and is currently classified as VUS by ACMG. As indicated, alterations in *TTN* are mainly associated with DCM, and the rare variant was also classified as VUS following ACMG recommendations. Neither parent was clinically affected, despite the mother continuously having a QTc of ~440 ms in several ECGs performed during follow-up of the index case. In addition, previous sudden death of a brother (II.1) at young age was documented with no conclusive cause of death after complete autopsy, with suspicions of arrhythmogenic death. However, no genetic analysis was performed, and no post-mortem sample was available. The most plausible cause of LQTS seems to be the rare variant in *KCNQ1* [previously reported in LQTS in a P role (CM040442) and currently also classified as LP by ACMG], but we cannot discard a potential pathogenic role of the novel deletion in *RyR2*—it is currently classified as P by ACMG and could play a key role in this family. *RyR2* has been associated with a minority of LQTS cases. The mother (I.2) carried both variants and could be considered as incomplete penetrance (42). In conclusion, this family does not show clear segregation, so the role of identifying rare variants remains to be clarified.

In the sixth family, four relatives were clinically diagnosed with LQTS (index case: III.1; family members: I.2, II.2, and II.4). The index case showed a maximum QTc of 485 ms. All other relatives showed QTc > 475 ms and treated with propranolol; no arrhythmogenic episodes have been documented in any of them to date. Genetic analysis identified four rare variants, with complete segregation of *ANK2_*p.Asp955His. This family had two premature deaths at young ages (II.3 and III.2, no definite cause of death after an autopsy). No DNA is currently available to perform molecular autopsy. Therefore, the rare variant in *ANK2* seems to be the most plausible cause of LQTS in this family, although it is currently classified as VUS following ACMG recommendations. Due to suspicious family history of inherited arrhythmia, this mother was referred at our center, but no complete assessment of relatives had been performed before inclusion in our study.

In family seven, only the index case (II.1) showed a prolonged QT interval of 510 ms. Propranolol was administrated and QTc value was reduced near to normality, and no arrhythmogenic episodes have been reported to date. Genetic analysis identified a rare variant in *SLC8A1*, inherited from his mother (I.2). Neither parent was clinically affected, and no family history of the disease or SCD was documented. This gene has been associated with delayed after-depolarizations leading to cardiac arrhythmias due to alterations in calcium concentrations. To date, no definite association with LQTS is reported. Therefore, the rare variant in *SLC8A1* does not seem to be the cause of disease in this family.

In family eight, two siblings were clinically diagnosed with LQTS (index case: II.2; and her sister: II.1). The index case had a QTc of 514 ms, and her sister had a maximum QTc of 485 ms. Propranolol was administered to both. No syncope or any arrhythmogenic episode has been reported to date. Neither parent showed any clinical symptoms related to LQTS. Genetic analysis identified three rare variants in *ANK2, SCN4B*, and *GAA*. One clinically diagnosed sibling only carried two rare variants in *ANK2* and *SCN4B*. The mother (I.2) carried only the *ANK2* variant, and the father (I.1) carried the other two variants in *SCN4B* and *GAA*. Both rare variants in *ANK2* and *SCN4B* are currently classified as LB following ACMG recommendations; therefore, none seems to be responsible for the disease. The third variant in *GAA* is currently classified as LP but is associated with Pompe disease and not LQTS. However, individuals in our study who carry this variant did not show phenotypes of Pompe disease (Figure 3; Table 2).

## Discussion

Infant health and control of infant mortality are among the priorities of healthcare systems and the World Health Organization (43). Neonates are at risk of arrhythmia due to immaturity of the cardiac conduction system and autonomic nervous system. Thus, early identification of neonates at risk of SADS and adoption of personalized therapeutic measures is crucial to reduce lethal episodes (17). In our study, ECG in a cohort of newborns identified 1.16% of cases with a potentially dangerous prolonged QT interval >470 ms, similar to recently reported (24). In this recent report, the authors reported 0.62% of patients with a QTc > 500 ms, also in concordance to our cohort. After follow-up, 0.43% of our cases were definitely diagnosed with LQTS. Previous studies reported percentages between 0.02 and 0.09% of certain LQTS (10, 23, 24). This higher percentage in our cohort may be because our institutions are national reference center for malignant arrhythmias and mothers with possible arrhythmogenic alterations during pregnancy and/or potential family history of malignant arrhythmias are referred to our center for assessment and follow-up. It is also important to remark that all families diagnosed with an LQTS in our cohort received first diagnosis at our institution but suspicious of inherited disease in relatives happened in four families. In consequence, if no inclusion of these four families due to referred at our center, after follow-up we identified 0.14% of cases with a definitely diagnosed with LQTS being more similar to previous studies and according to widely-accepted prevalence of LQTS in global population. At our point of view, this is a crucial point in the interpretation of data because the prevalence of LQTS in our country is not higher in comparison to other. The discrepancy about higher prevalence in neonates is due to receiving families with suspicious inherited arrhythmia despite not conclusive diagnosis. Taking all this data together, our results, in agreement with these previous studies, support that ECG screening of newborns is a successful approach to identifying prolonged QT intervals despite being a rare inherited disease.

LQTS is an established inherited genetic disease, so current guidelines recommend both clinical and genetic analyses in relatives (16, 30). In our study, comprehensive genetic analysis of all genes currently associated with LQTS identified a potential pathogenic variant in 62.5% of newborns with a prolonged QT interval, accordingly to 80–85% of the genetic yield after a comprehensive genetic analysis of all genes currently associated with LQTS (13). In addition, familial genotype–phenotype assessment of newborns with a prolonged QT interval identified at least one clinically affected relative in all families, and 36.6% of analyzing relatives were diagnosed with LQTS, also similar to previous reports (44).

At present, the inclusion of the ECG in neonatal assessment is a matter of debate, despite ECG is a non-invasive and feasible tool to assess the cardiac conductive system and that QT alterations are a reliable indicator to predict susceptibility to cardiac arrhythmia and risk of SCD (45). From our perspective, studies performed so far highlight many arguments supporting the use of the ECG in newborns (10, 23, 24). Prolonged QT intervals account for at least 10% of SIDS, screening is feasible and, perhaps most importantly, arrhythmia is largely treatable, reducing the risk of lethal episodes. However, the key point in discussion concerns false positive results in the first hours of life (46). This can lead to unnecessary anxiety of parents and even unnecessary therapy (47). False positives are due to continuous changes in the ECG of a newborn (48). This fact did not occur in our study due ECG was repeated in each case with a potential dangerous QTc value, at different times after birth. A proper ECG interpretation is crucial and may affect patient care, especially in neonates. As recently described, accuracy among pediatric cardiologists is higher than in pediatricians in the interpretation of the ECG in neonates, and both groups recognize the importance of ECGs at early age (49). Our approach is in concordance with recent results supporting Bazett's formula as an appropriate method for correction of the QT interval for elevated sinus heart rates of newborns (26) and stable value during the first month of life (27). Further, close follow-up and personalized therapeutic measures should be adopted according to current guidelines (16). In our study, and as performed in other previous studies (10, 23, 24), continuous ECG during the first months of life in all newborns with suspicious QTc (>450 ms) can clarify if ECG alterations are a sign of LQTS or only electric modifications due to physiological adaptation. Our results show that only newborns with QTc > 480 ms in the first hours of life maintain prolonged QTc values in follow-up ECGs and are at high risk of SCD, in concordance to previous studies of 1-month-old infants (23).

Our comprehensive genetic analysis identified at least one rare variant in all families, but a conclusive family segregation was observed in only three cases, supporting a deleterious role of the rare variant as the main responsible for LQTS. These rare variants are classified as LP or P and are located in one of three primary genes definitively associated with LQTS (14, 50). Hence, the yield of genetic testing of clinically diagnosed patients with QTc ≥ 470 ms is low, which can be due to false positive cases misdiagnosed as LQTS or current unknown roles of rare variants classified as VUS (51). At our point of view, classification as VUS does not mean that there is a less pathogenic risk of LQTS for any patient who carries the rare variant; instead, ambiguous significance implies that current evidence does not provide a conclusive deleterious role. Therefore, clinical translation of VUS should be performed with caution by experts, as recently recommended (50), and VUS should not be discarded until additional data can definitively clarify a clinical role. Current ACMG/AMP recommendations for interpretation of rare variants include a large list of items that make the classification more accurate, but also thus more stringent (34), so lack of data for some of these items can lead to ambiguous classification (52). Comprehensive evaluation of clinical findings and pathogenicity of variants based on ACMG/AMP-based evaluation may stratify arrhythmic risk of LQTS (53). Discriminating a true risk-carrying variant from a non-deleterious variant is a challenge without accurate family segregation and functional studies. Concerning genetically positive cases, no *de novo* variants were identified in our study despite another report showed that ~12–15% of newly identified patients with LQTS have *de novo* alterations (54). This could be due to the small number of cases affected by LQTS. This limited number of cases could also explain why no CNVs were identified in our cohort despite it is reported that 3–5% of LQTS cases can be caused by CNVs (33, 55, 56).

In addition, due to an inherited disease, familial screening was also performed in all available cases, according to current guidelines (16, 30, 57, 58). In our center we perform a close follow-up of all families diagnosed with any arrhythmogenic disease, especially if high risk of SCD is present in any the relatives. Family segregation can help clarify the role of rare genetic variants identified in newborns as well as enable early identification of relatives carrying potentially pathogenic genetic alterations, who are thus at risk of SCD. Hence, after genotype–phenotype correlation, 62.5% of cases carried a highly potentially deleterious variant, similar to previous comprehensive genetic studies performed in LQTS cohorts (38).

### Limitations

Our study has some limitations that should be noted. Despite previous published study in a large cohort (4), additional studies in large cohorts of different hospitals and countries are required to obtain a more representative prevalence of prolonged QT interval in the neonatal population. Moreover, our results cannot be used to assess true LQTS prevalence in the non-Caucasian population. Our study also lacks analysis of other genes not included in our NGS custom panel and that could be implicated in SADS. A potential future approach is to perform whole exome sequencing and/or whole genome sequencing to identify new alteration in any region of the genome. Family segregation is a key point for definite classification of genetic variants of ambiguous significance; in addition, functional studies of each genetic variant may help clarify the associated pathophysiological mechanism. Following this point, classification of rare variants should be done following ACMG/AMP recommendations and periodically should be reanalysed, especially if classified as VUS. A periodic update of previous classification may help to clarify role of rare variants, helping to clinicians to obtain genetic diagnosis and, if appropriate, adopt preventive measures. It is also important to highlight the necessity of molecular autopsy in cases with suspicious inherited arrhythmia. In our study, as occurs in other centers, large part of families with cases of sudden death without conclusive diagnosis, neither DNA or tissue sample are available for genetic study. Current forensic protocols recommend inclusion of molecular autopsy in these cases but unfortunately, it is not performed as routine in most part of forensic centers, at least in our country. Finally, it is important to note that although ECG is a simple and low-cost approach, medical costs differ in each country, so a comprehensive cost-effective study should be done before implementation.

## Conclusions

In summary, ECG screening in infancy has not yet been implemented in any European country. Our results support implementation of the ECG in neonatal screening protocols as a feasible and non-invasive tool to assess QT interval as a consistent indicator to predict susceptibility to cardiac arrhythmia. Despite a low percentage of infants showing an enlarged QT interval after close follow-up (0.14%), performing ECGs in during the neonatal period is an effective approach to identify newborns at risk of SADS. Comprehensive genetic analysis in cases showing a prolonged QTc of >480 ms can unravel the cause of disease in most cases. Further, relatives can also benefit from clinical and genetic analyses of these inherited diseases in neonates. Despite genetic variant interpretation, remaining a current challenge prior to clinical translation, identifying genetic carriers allows adoption of personalized preventive measures to reduce risk of arrhythmias and SCD.

## Data Availability Statement

The original contributions presented in the study are included in the article/supplementary materials, further inquiries can be directed to the corresponding authors.

## Ethics Statement

The studies involving human participants were reviewed and approved by Hospital Josep Trueta−2015/053-, Hospital Sant Joan de Déu -PIC/98/15-, Hospital del Mar−2015/6250/1-. Written informed consent to participate in this study was provided by the participants' legal guardian/next of kin.

## Author Contributions

GS-B, OG-A, OC, and RB developed the concept and prepared the manuscript. MZ, AF-F, SS, SC GS, JM-A, EAu, JC, EM, MC, AP-S, BO, VF, AI, CF-C, MP, LL, FP, EAr, and PJ acquired, pre-processed, and analyzed the data. GS-B, OG-A, JB, OC, and RB supervised the study. All authors contributed to manuscript revision, read, and approved the submitted version.

## Conflict of Interest

The authors declare that the research was conducted in the absence of any commercial or financial relationships that could be construed as a potential conflict of interest.

## Publisher's Note

All claims expressed in this article are solely those of the authors and do not necessarily represent those of their affiliated organizations, or those of the publisher, the editors and the reviewers. Any product that may be evaluated in this article, or claim that may be made by its manufacturer, is not guaranteed or endorsed by the publisher.
